# Oral Administration of Propolis and Lysozyme Combination Improves Feline Oral Health and Modulates Systemic Inflammatory and Oxidative Responses

**DOI:** 10.3390/antiox14060639

**Published:** 2025-05-26

**Authors:** Longjiao Wang, Qianqian Chen, Weiwei Wang, Hao Dong, Xiaohan Chang, Lishui Chen, Ran Wang, Yaoxing Chen, Pengjie Wang, Shuxing Chen, Wei Xiong, Yixuan Li

**Affiliations:** 1 Key Laboratory of Precision Nutrition and Food Quality, Department of Nutrition and Health, China Agricultural University, Beijing 100083, China; longjiaowang2019@163.com (L.W.); wangran@cau.edu.cn (R.W.); wpj1019@cau.edu.cn (P.W.); 2 Food Laboratory of Zhongyuan, Luohe 462300, China; chenqianqian@zyfoodlab.cn (Q.C.); bingzhi213608@163.com (W.W.); donghao980623@163.com (H.D.); changxiaohan@zyfoodlab.cn (X.C.); chlishui@sina.com (L.C.); chenshuxing1@163.com (S.C.); 3 College of Veterinary Medicine, China Agricultural University, Beijing 100193, China; yxchen@cau.edu.cn

**Keywords:** feline, oral microbiota, propolis, lysozyme, oral health, inflammatory, oxidative responses

## Abstract

Oral diseases are highly prevalent among domestic cats, with microbiota dysbiosis as a primary etiological factor. However, effective microbiota-targeted interventions remain limited. This study evaluated the efficacy of a dietary supplement combining propolis and lysozyme (PL) in mitigating feline oral health issues, based on a cohort of 24 cats divided equally into placebo, treatment, and healthy control groups (*n* = 8 per group). Supragingival microbiota were analyzed via 16S rRNA gene sequencing, alongside assessments of volatile sulfur compounds (VSCs), oral health indices, and systemic inflammatory, oxidative, and immune markers. After 28 days of intervention, cats receiving PL supplementation demonstrated significant improvements, including a 35.4% reduction in VSCs and notable decreases in debris (34.9%), plaque (51.2%), and gingival indices (61.0%). Systemically, MDA and TNF-α levels decreased, while SOD, T-AOC, and IL-4 increased. Microbiota analysis revealed suppression of Porphyromonas and Selenomonas and enrichment of Moraxella and Bergeyella. Reductions in VSCs, gingival index, and TNF-α were correlated with lower Porphyromonas abundance, while Moraxella and Luteimonas were positively associated with antioxidant status. Functional predictions indicated downregulation of virulence-related pathways and increased expression of glutathione reductase. These findings highlight PL’s potential as a natural, microbiota-based intervention that improves feline oral health and modulates the oral–systemic axis, supporting its application in integrative oral care strategies.

## 1. Introduction

Oral diseases represent one of the most common health challenges in domestic cats, with over 70% developing periodontal disorders by the age of 2 [1]. Halitosis frequently serves as the earliest clinical manifestation, followed by the accumulation of dental plaque, calculus formation, and gingivitis. Without timely intervention, these conditions may progress to periodontitis, leading to pain, tooth loss, and feeding impairments [2]. Beyond localized pathology, increasing evidence suggests that oral inflammation contributes to systemic immune activation and oxidative stress, potentially exacerbating overall physiological dysfunction [3].

As the second most diverse microbial ecosystem in cats after the gut, the oral microbiota plays a pivotal role in sustaining oral and systemic homeostasis [4]. Volatile sulfur compounds (VSCs), such as hydrogen sulfide (H_2_S) and methyl mercaptan (CH_3_SH), are key contributors to halitosis and are primarily produced by anaerobic bacteria, including *Porphyromonas gingivalis*, *Prevotella intermedia*, and *Fusobacterium nucleatum* [5,6]. These bacteria also secrete lipopolysaccharides (LPSs), gingipains, and other virulence factors that disrupt gingival tissue integrity and promote biofilm formation. Mounting evidence indicates that pathogenic oral microbes and their metabolites may translocate across compromised oral epithelial barriers, triggering systemic inflammatory and oxidative responses in distal organs [7]. For example, oral microbial transfer from diabetic mice has been shown to aggravate periodontal inflammation and upregulate systemic IL-6 and RANKL expression [8].

Modulating the oral microbiota is increasingly recognized as an effective strategy for controlling both oral and systemic inflammation. While antiseptics such as chlorhexidine remain widely used in veterinary settings, prolonged use may disrupt microbial homeostasis and lead to adverse outcomes, including mucosal irritation and tooth discoloration [9]. However, natural antimicrobial agents, including polyphenols and bioactive peptides, offer safer alternatives with favorable tolerability and broad-spectrum efficacy. For example, catechin has been shown to reduce malodor in cats [10], while Chenopodium murale leaf demonstrated therapeutic effects against oral candidiasis in rats, along with a significant reduction in serum markers of oxidative stress—malondialdehyde (MDA) [11].

Lysozyme is an endogenous antimicrobial enzyme present in feline saliva that selectively hydrolyzes the peptidoglycan layer of Gram-positive bacteria, disrupting plaque biofilms and promoting mucosal immune balance [12,13]. However, Gram-negative species—particularly those responsible for VSC production—are less susceptible to lysozyme due to their protective outer membrane structure [14,15]. Propolis, a resinous compound produced by bees, has demonstrated antibacterial activity against Gram-negative pathogens via mechanisms including membrane disruption [16,17], interference with nucleic acid synthesis, and downregulation of virulence gene expression [18,19,20]. Moreover, propolis is rich in flavonoids and phenolic acids that exert systemic antioxidant and anti-inflammatory effects.

Despite evidence supporting the individual benefits of lysozyme and propolis, their synergistic potential in modulating both oral and systemic physiology remains largely unexplored, particularly in companion animals. Thus, we investigated the combined effects of propolis and lysozyme (PL) on oral health, microbial ecology, and systemic redox-inflammatory status in domestic cats. We hypothesized that PL intervention would improve halitosis and oral inflammation through microbial modulation, while also conferring systemic benefits via attenuation of oxidative stress and immune dysregulation. Through integrated analyses of oral symptoms, serum biomarkers, and microbiome structure and function, this study aims to provide mechanistic insight into the therapeutic potential of natural antimicrobial agents in feline health.

## 2. Materials and Methods

### 2.1. Experimental Animals and Experimental Design

Sixteen privately owned domestic cats clinically diagnosed with halitosis (VSCs ≥ 500 ppb) were randomly allocated to either the placebo or treatment group (*n* = 8 per group). An additional cohort of eight healthy cats (VSCs < 500 ppb) was included as a control group to establish baseline oral microbiota composition. Cats with recent exposure to antimicrobial or probiotic agents, underlying chronic systemic diseases, or recent oral treatments were excluded from participation.

The intervention trial lasted 28 days and was conducted in accordance with the ethical guidelines of the China Agricultural University (Protocol No. AW60704202-5-2). Detailed demographic characteristics of the animals, including age, sex, and body weight, are summarized in Table 1.

Cats in the treatment group received 2 g/day of freeze-dried chicken cubes supplemented with 0.3% propolis (Solepol, Beijing, China) and 0.2% lysozyme (Aladdin, Shanghai, China). Cats in the placebo and healthy control groups received unsupplemented chicken cubes. All animals were fed the same standard basal diet and were not provided with additional functional supplements throughout the study period.

### 2.2. Measurement of VSC Concentration

VSC levels were assessed on days 0, 7, 14, 21, and 28, between 8:00 and 9:00 a.m. To minimize variability due to recent feeding, all cats were fasted for at least 2 h prior to sampling. A Halimeter breath analyzer (Interscan Corp., Chatsworth, CA, USA) was used to measure gas from the cats’ oral cavity. All procedures were performed in accordance with the manufacturer’s instructions. Each animal underwent three independent measurements, and the final VSCs’ value was calculated as the average of the three replicates. In addition, healthy cats underwent the same measurement procedure to establish baseline reference values.

### 2.3. Cat Supragingival Flora Collection

Following the VSC measurements (between 8:00 and 9:00 a.m.), supragingival plaque samples were collected by gently swabbing the gingival margins of the cats’ premolar teeth using sterile cotton swabs for 30 s. Each swab head was then transferred into a 10 mL sterile centrifuge tube containing 750 μL of MoBio lysis buffer (Mo Bio Laboratories, Carlsbad, CA, USA) and stored at −80 °C until further analysis.

### 2.4. Oral Symptoms Indices

Oral health assessment was conducted in accordance with a simplified scoring protocol adapted from the “Pet Smile Preventive Measures Activity”, which aligns with standard veterinary dental diagnostic practices. Evaluated parameters included dental debris, plaque, calculus, and gingival inflammation. The following established indices were used for scoring: the Turesky–Gilmore–Glickman modification of the Quigley–Hein Plaque Index (PLI), the Ramfjord Calculus Index (CI), the modified Löe and Silness Gingival Index (GI), and the Greene–Vermillion Debris Index (DI). Assessments were performed on days 0, 14, and 28 by six trained evaluators. The scoring focused on the following teeth: maxillary I3, C, P3, P4, M1, and mandibular C, P3, P4, M1. In addition, healthy cats underwent the same scoring procedure to provide baseline reference values.

### 2.5. 16S rRNA Sequencing of Supragingival Samples

Total microbial DNA was extracted from supragingival plaque samples using the E.Z.N.A.^®^ Soil DNA Kit (Omega Bio-tek, Norcross, GA, USA) in accordance with the manufacturer’s protocol. DNA quality and concentration were evaluated by agarose gel electrophoresis and NanoDrop 2000 spectrophotometer (Thermo Fisher Scientific, Waltham, MA, USA). The V3–V4 hypervariable region of the 16S rRNA gene was amplified using primers 338F and 806R and sequenced on the Illumina MiSeq platform (Illumina, San Diego, CA, USA) by Majorbio Bio-Pharm Technology Co., Ltd. (Shanghai, China). Library construction was performed using the NEXTflex™ Fast DNA-Seq Kit (Bioo Scientific, Austin, TX, USA). Raw sequencing reads were quality-filtered and trimmed using fastp (version 0.20.0). Operational taxonomic units (OTUs) were clustered using UPARSE, and taxonomic classification was conducted using the Ribosomal Database Project (RDP) classifier.

### 2.6. Blood Collection and Testing

On day 28 of the intervention, venous blood was collected from the forelimbs of the cats by a licensed veterinarian. Serum samples were not collected from the healthy control group in order to minimize invasive procedures and animal stress, as per ethical guidelines. Healthy cats served primarily as non-invasive phenotypic and microbial references; 1.5 mL of whole blood was allowed to clot at room temperature for 2 h, followed by centrifugation at 3000× *g* for 15 min. The resulting serum was separated and stored at −80 °C until further use. Serum immunological, inflammatory, and antioxidant biomarkers were quantified using commercial ELISA kits (MMBio, Shanghai, China) according to the manufacturer’s instructions.

### 2.7. Statistical Analysis

Statistical analyses were performed using GraphPad Prism software (version 9.0, GraphPad Software Inc., San Diego, CA, USA). All results are presented as mean ± standard deviation (SD). Group differences were assessed using Student’s *t*-test or one-way analysis of variance (ANOVA), as appropriate. A *p*-value < 0.05 was considered statistically significant. Levels of significance were denoted as *p <* 0.05, *p <* 0.01, and *p <* 0.001.

The 16S rRNA gene sequences were analyzed using the Majorbio Cloud Platform (https://www.majorbio.com (accessed on 23 May 2025)). Taxonomic and association analyses were performed as follows. Taxonomic abundance at different levels was assessed through community composition analysis, and species-level abundance was visualized using bar plots. Principal Coordinate Analysis (PCoA) was conducted to evaluate beta diversity among groups. Key discriminant taxa were identified using Linear Discriminant Analysis Effect Size (LEfSe), while Linear Discriminant Analysis (LDA) was applied to estimate the relative contribution of specific species and predicted functions to group differences. Species associated with halitosis were selected for differential abundance analysis. Correlations between microbial taxa and both oral health indices and systemic physiological parameters were assessed using Spearman’s correlation coefficient and visualized as heatmaps. Functional predictions were performed using the Tax4Fun algorithm, which maps 16S rRNA profiles to KEGG functional pathways based on taxonomic assignments and reference gene catalogs from the Ref100NR database, including curated microbial metabolic pathway annotations.

## 3. Results

### 3.1. Baseline Characteristics of Participating Cats

At the beginning of the study, no significant differences were observed in baseline characteristics among the three groups—including age, sex, and body weight, indicating successful randomization (Table 1). The concentrations of VSCs were significantly different between halitotic and healthy cats (*p* < 0.001), while almost no difference between the treatment and placebo groups (*p* = 0.9669).

### 3.2. Improvement of VSCs in Cats with Halitosis by PL Intervention

**Figure 1:**
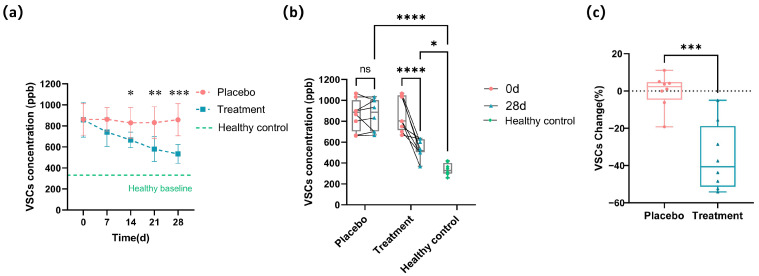
Changes in the VSCs’ profile after 28 days of intervention. (**a**) Longitudinal change in the overall VSC concentrations (ppb) in the placebo and treatment groups during the 28-day intervention, with a dashed line indicating the mean VSC level in healthy cats. (**b**) Individual VSC concentrations for each cat at baseline (0 days) and after 28 days of intervention. Healthy baseline values are shown as green reference points. (**c**) Percentage change in VSC concentration at the end of the experiment relative to the starting point for each cat in the placebo and treatment groups (* *p <* 0.05, ** *p* < 0.01, *** *p* < 0.001, **** *p* < 0.0001).

The changes in VSC concentrations for the two groups of cats are illustrated in Figure 1a. Initially, the VSC levels in the placebo and experimental groups were 861.67 ± 149.74 ppb and 852.08 ± 157.72 ppb, respectively, with no statistically significant difference (*p* ≥ 0.05). After 14 days of intervention, the VSC level in the treatment group decreased to 666.67 ± 73.46 ppb, which was significantly lower than that in the placebo group (829.17 ± 145.23 ppb; *p <* 0.05), representing a 19.6% reduction compared to the placebo group. By day 28, the VSC level in the placebo group remained at 858.33 ± 150.78 ppb, whereas that in the treatment group dropped further to 533.33 ± 89.09 ppb, with a highly significant difference between the groups (*p <* 0.001), reflecting a 37.8% decrease in the treatment group compared to the placebo. Figure 1a includes a horizontal dashed line indicating the average VSC level observed in healthy cats (332.38 ppb), serving as a visual baseline reference. The treatment group showed a continuous reduction in VSC levels approaching the healthy baseline by day 28, whereas the placebo group showed minimal change, remaining markedly elevated. Figure 1b displays the individual VSC concentration profiles for each cat on days 0 and 28, revealing minor fluctuations in the placebo group, while the treatment group displayed a general decline from baseline. To facilitate comparison, baseline VSC levels from healthy cats (*n* = 8) were included in Figure 1b. By day 28, the treatment group showed VSC concentrations that were significantly lower than the placebo group (*p* < 0.001), and although still higher than the healthy baseline (*p* < 0.05), they demonstrated a marked trend toward normalization. The relative changes in VSC concentrations from baseline were calculated for each cat. As shown in Figure 1c, the placebo group exhibited a negligible change in VSC concentrations (+0.71%), whereas the treatment group showed a substantial decrease of −35.37%.

### 3.3. Oral Symptom Improvement in Response to PL Supplementations

**Figure 2:**
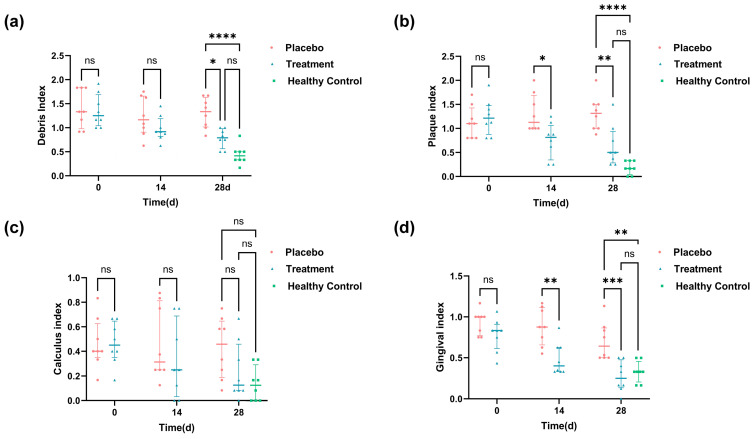
Oral health scores and visual records of oral symptoms in cats throughout the experiment: (**a**) DI scores at 0, 14, and 28 days; (**b**) PI scores on days 0, 14, and 28; (**c**) CI scores at 0, 14, and 28 days; and (**d**) GI scores in 0, 14, and 28 days. Healthy control values are shown as baseline references (* *p* < 0.05, ** *p* < 0.01, *** *p* < 0.001, **** *p* < 0.0001).

In this study, we evaluated several oral symptom indices both before and after the 28-day intervention, including PLI, CI, DI, and GI. The results are presented in Figure 2. Figure 2a shows no significant difference in the initial DI between the placebo and treatment groups. However, after 28 days of intervention, the treatment group showed a notable 34.9% decrease in DI compared to the placebo group. Regarding plaque control, the PLI indicated a 51.2% reduction in the treatment group compared to that in the placebo group after 28 days of intervention (Figure 2b). Dental calculus is formed by plaque mineralization; in terms of dental calculus, there was no significant improvement in CI after 28 days of intervention (Figure 2c), indicating that the PL intervention had limited effects on calculus formation over the study period. For gingival health, the GI demonstrated a significant improvement. After just 14 days of intervention, the GI showed a striking 37.33% reduction in the treatment group compared to the placebo group, with this improvement increasing to 61.04% by day 28 (Figure 2d). By day 28, scores for DI, PLI, and GI in the treatment group were statistically indistinguishable from those of healthy controls (*p* > 0.05), indicating a near-complete restoration of oral health.

### 3.4. Effects of Propolis and Lysozyme Ingestion on Immune Responses, Oxidative Stress Levels, and Inflammatory Markers in Domestic Cats

Improvements in oral health are often accompanied by systemic benefits, including modulation of immune responses, reduction in inflammation, and alleviation of oxidative stress. In this study, serum biomarkers were analyzed to assess these systemic effects.

**Figure 3:**
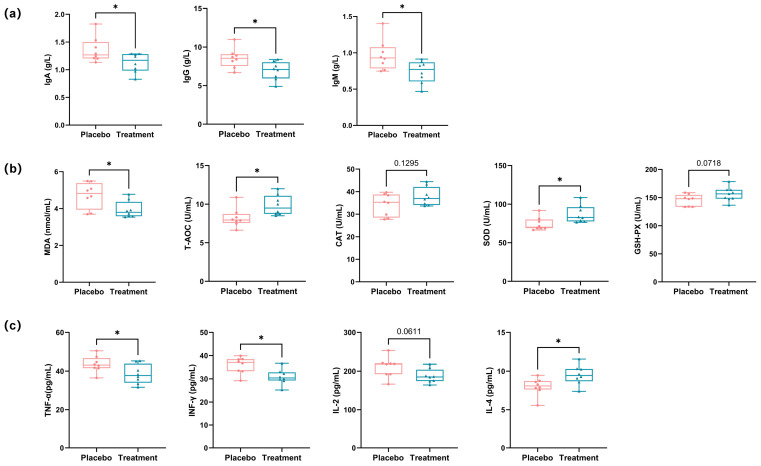
Analysis of immune responses, oxidative stress levels, and inflammatory markers in cat serum. (**a**) Analysis of immune responses in cat serum, showing changes in immunoglobulin levels (IgA, IgG, IgM) (* *p <* 0.05). (**b**) Analysis of oxidative stress levels in cat serum, showing changes in MDA, T-AOC, CAT, SOD, and GSH-PX (* *p <* 0.05). (**c**) Analysis of inflammatory markers in cat serum, showing changes in TNF-α, IFN-γ, IL-4, and IL-2 (* *p <* 0.05). IgA: immunoglobulin A; IgG: immunoglobulin G; IgM: immunoglobulin M; MDA: malondialdehyde; SOD: superoxide dismutase; T-AOC: total antioxidant capacity; TNF-α: tumor necrosis factor alpha; IFN-γ: interferon gamma; IL-4: interleukin-4; IL-2: interleukin-2.

PL supplementation resulted in a significant decrease in systemic immunoglobulin levels. Specifically, Immunoglobulin A (IgA), Immunoglobulin G (IgG), and Immunoglobulin M (IgM) levels dropped by 16.9%, 19.2%, and 23.7%, respectively, compared to the placebo group (*p* < 0.05 for all), indicating a reduction in antigenic stimulation or mucosal immune activation (Figure 3a). Oxidative stress markers were also modulated. Malondialdehyde (MDA), a lipid peroxidation product, was reduced by 16.4% in the PL group (*p* < 0.05). In addition, key antioxidant markers—superoxide dismutase (SOD) and total antioxidant capacity (T-AOC)—were significantly elevated, supporting improved systemic redox balance. Catalase (CAT) and glutathione peroxidase (GSH-PX) showed mild, non-significant increases (Figure 3b). Pro-inflammatory cytokines followed a similar trend. Tumor necrosis factor-α (TNF-α) and interferon-γ (IFN-γ) levels were significantly lower in the PL group, while interleukin-2 (IL-2) showed a slight but non-significant decline; whereas interleukin-4 (IL-4), an anti-inflammatory cytokine, increased by approximately 22.3%, suggesting a shift toward immune regulation and homeostasis (Figure 3c).

### 3.5. Taxonomic Composition of Cat Gingival Microbiome

**Figure 4:**
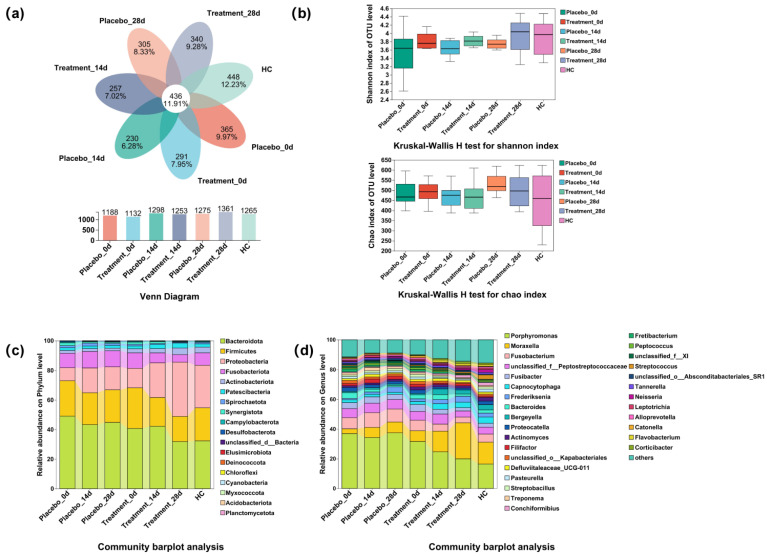
Changes in diversity and species composition of the supragingival microbiome after 28 days of intervention. HC, healthy control. (**a**) Venn diagram showing the overlap of OTUs across the different groups. (**b**) Kruskal–Wallis H test comparing Shannon and Chao diversity indices between groups. (**c**) Microbiota composition at the phylum level for each group. (**d**) Microbiota composition at the genus level for each group.

To gain a deeper understanding of how PL influences the composition of feline gingival microbiome, we collected supragingival flora samples from both the halitosis and healthy control groups and conducted 16S rRNA sequencing. Following quality control optimization, comprehensive taxonomic identification was performed. A total of 3661 OTUs were identified in the supragingival samples from each group. Of which 436 OTUs were present in all groups, indicating the presence of core microbiota composition (Figure 4a). We assessed the taxonomic diversity using α-diversity indices: Chao and Shannon. The analysis did not differ significantly among the groups (*p* ≥ 0.05), suggesting that PL treatment did not significantly alter overall microbial diversity (Figure 4b). We identified 824 species belonging to 380 genera within 20 phyla, 43 orders, 95 families, and 171 families. At the phylum level, *Bacteroidetes* was the most dominant, followed by *Firmicutes*, *Proteobacteria*, *Fusobacteria*, *Actinobacteria*, and *Patescibacteria* (Figure 4c). At the genus level, *Porphyromonas* was the most abundant, followed by *Moraxella*, *Fusobacterium*, *Peptostreptococcaceae*, *Fusibacter*, and *Frederiksenia* (Figure 4d).

These results highlight the complex microbial composition of the feline oral cavity and suggest that PL treatment may influence specific taxa, though no significant changes in microbial diversity were observed. The core microbiota shared across all groups provides insights into stable oral microbial features, while differences in the relative abundance of key genera warrant further exploration.

### 3.6. Changes in the Distribution of Microbiome on the Gingiva of Cats with Halitosis Associated with PL Intervention

**Figure 5:**
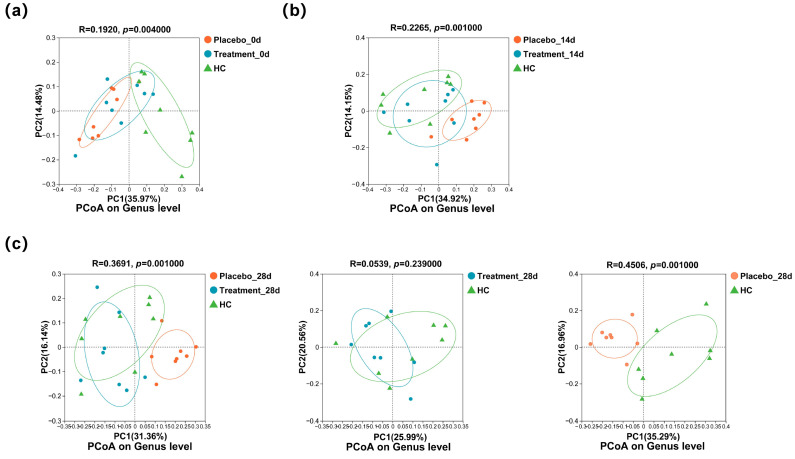
Changes in the genus composition of the supragingival microbiome of cats during the 28 days of intervention: (**a**) PCoA of the three groups at the genus level on day 0; (**b**) PCoA of the three groups at the genus level on day 14; and (**c**) PCoA of the three groups at the genus level on day 28.

To assess whether PL intervention affected the composition of the oral microbiota, PCoA was performed. At baseline (Figure 5a), the supragingival microbial communities of the treatment and placebo groups were comparable, yet both differed significantly from those of the healthy control (HC) group (*p* < 0.05). After 14 days of intervention (Figure 5b), the microbiota compositions of the treatment and placebo groups began to diverge. By the end of the study (Figure 5c), the microbiota of the placebo group remained significantly different from that of the HC group (*p* < 0.05), whereas the microbial community of the treatment group showed no significant difference compared to the HC group (*p* ≥ 0.05). These findings suggest that PL supplementation contributed to the restoration of a healthier oral microbiome composition.

### 3.7. Characteristic Taxa Variation in the Gingival Microbiome in Cats with Halitosis

**Figure 6:**
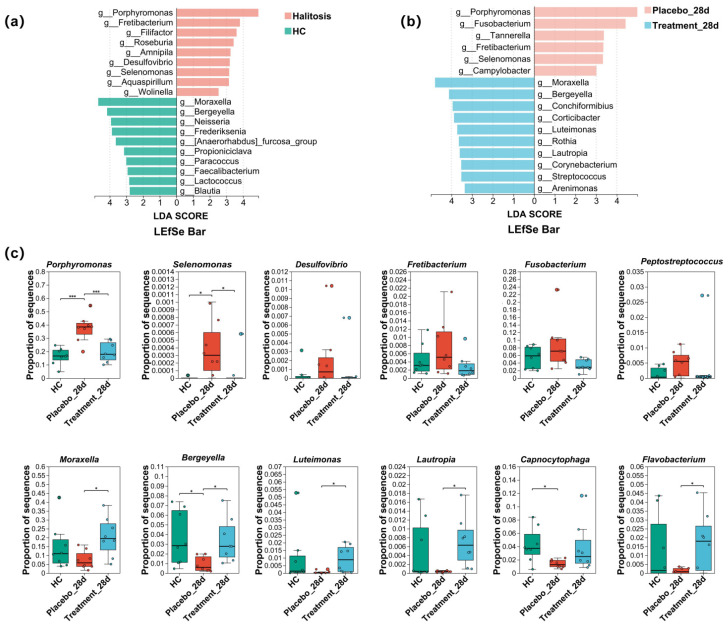
Effects of PL intervention on abundance changes in characteristic taxa at the genus level. (**a**) Taxonomic cladogram from LEfSe analysis showing differences in genus-level composition between the healthy control and treatment groups. (**b**) Taxonomic cladogram from LEfSe analysis showing differences in genus-level composition between the placebo and treatment groups after PL intervention. (**c**) Comparison of the relative abundance of 12 characteristic genera across the three groups (* *p <* 0.05, *** *p <* 0.001).

To identify microbial taxa associated with halitosis and healthy states, LEfSe analysis was performed, revealing 19 discriminative genera. Notably, *Porphyromonas*, *Fretibacterium*, *Filifactor*, *Roseburia*, *Desulfovibrio*, and *Selenomonas* were enriched in cats with halitosis, whereas *Moraxella*, *Bergeyella*, *Neisseria*, *Frederiksenia*, *Lactococcus*, and *Blautia* were predominant in healthy controls (Figure 6a). After 28 days of PL intervention, LEfSe analysis identified 16 taxa with differential abundance between the treatment and placebo groups. In the placebo group, genera such as *Porphyromonas*, *Fusobacterium*, *Fretibacterium*, *Selenomonas*, and *Campylobacter* remained dominant, while in the treatment group, *Moraxella*, *Bergeyella*, *Luteimonas*, and *Lautropia* were more prominent (Figure 6b).

Statistical comparisons (Figure 6c) revealed that the relative abundance of *Porphyromonas* was significantly higher in the placebo group than in healthy controls (*p* < 0.01). However, following PL supplementation, *Porphyromonas* levels in the treatment group significantly decreased to 19.47% ± 7.32%, approaching those observed in the healthy control group (16.48% ± 6.22%; *p* ≥ 0.05). Similarly, the relative abundances of *Selenomonas*, *Desulfovibrio*, *Fretibacterium*, and *Fusobacterium* were elevated in the placebo group but significantly suppressed after PL treatment. Notably, *Bergeyella*, which was significantly reduced in halitosis cats compared to healthy controls (*p* < 0.05), and *Moraxella*, which displayed a downward trend, both showed significant recovery post-intervention (*p* < 0.05), reaching levels comparable to healthy cats. In addition, the proportions of *Luteimonas*, *Lautropia*, and *Flavobacterium* were also significantly restored in the treatment group following PL administration (*p* < 0.05).

### 3.8. Correlation Between Oral Microbiota Composition and Systemic Physiological Indicators

**Figure 7:**
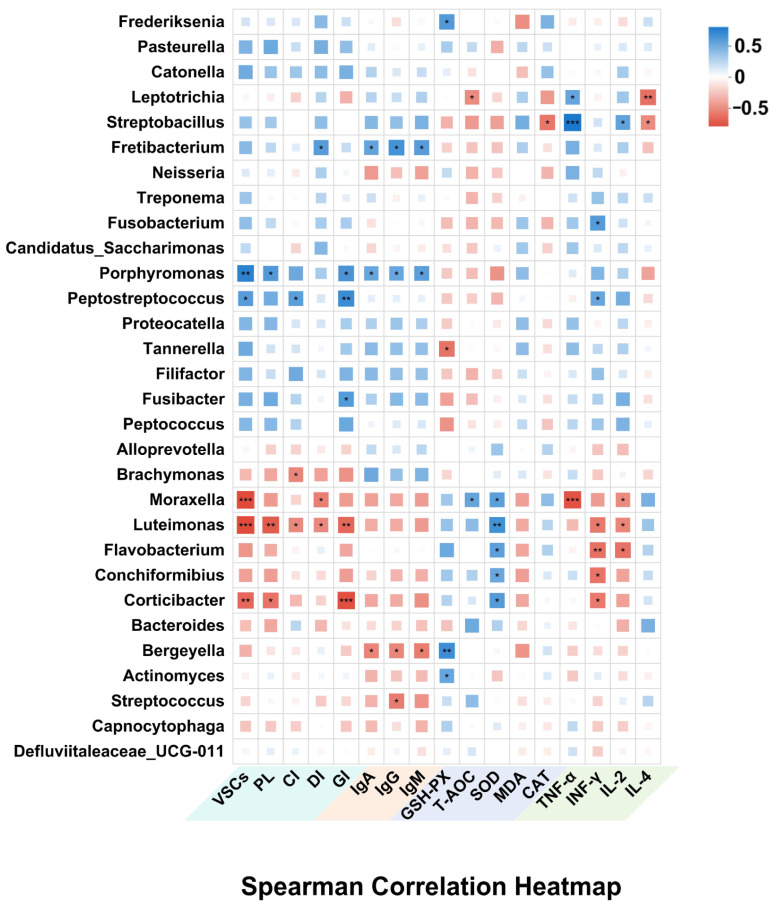
Analysis of the correlation between oral microbiome and oral, as well as overall health (* *p <* 0.05, ** *p <* 0.01, *** *p <* 0.001).

Spearman correlation analysis revealed significant associations between dominant oral bacterial genera and feline oral health indicators (Figure 7). Pathogenic taxa such as *Porphyromonas* and *Peptostreptococcaceae* were positively correlated with VSCs, PLI, and GI (*p <* 0.05), supporting their established role in halitosis and gingival inflammation. Additionally, *Fusobacterium* and *Fretibacterium* showed positive correlations with GI (*p <* 0.05), suggesting involvement in periodontal tissue degradation and biofilm maturation. Conversely, commensal genera such as *Moraxella*, *Luteimonas*, and *Corticibacter* were extremely significantly negatively correlated with VSCs and oral symptom indices (*p <* 0.01), indicating a potential role in maintaining oral microbial balance and mitigating oral symptoms.

Beyond local effects, several oral microbiota genera were also linked to systemic markers of immunity, inflammation, and oxidative stress. *Porphyromonas*, *Streptobacillus*, *Fusobacterium*, and *Leptotrichia* were positively correlated with MDA, TNF-α, and IFN-γ, while showing negative correlations with antioxidant enzymes, including SOD, GSH-PX, and T-AOC, as well as the Th2-type anti-inflammatory cytokine IL-4; these pathogenic genera may contribute to systemic oxidative stress and inflammatory responses. In contrast, commensal bacteria such as *Moraxella*, *Luteimonas*, and *Flavobacterium* showed strong positive correlations with SOD and T-AOC, and negative correlations with MDA, TNF-α, IFN-γ, and IL-2, indicating potential antioxidant and anti-inflammatory effects. Additionally, *Bergeyella* and *Streptococcus* were positively associated with IgG. These results emphasize the interconnectedness between oral microbiota and systemic health, highlighting the broader impact of oral microbial shifts on both oral health and systemic immunity, oxidative stress, and overall physiological status.

### 3.9. Functional Prediction of Oral Microbiota via Tax4Fun

**Figure 8:**
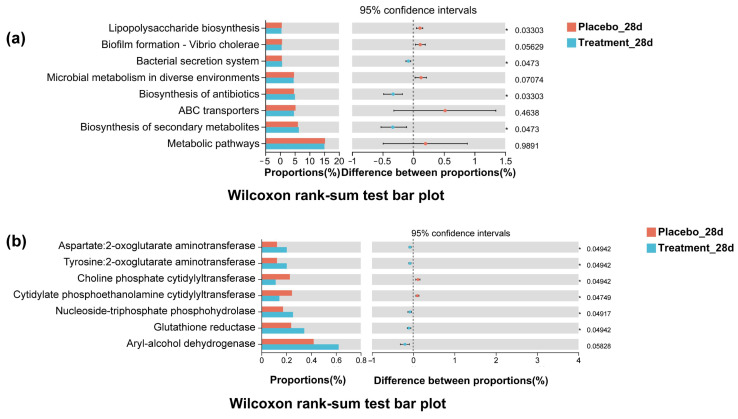
Tax4Fun-predicted functional differences between placebo and PL groups on day 28. (**a**) KEGG pathway differences, including reduced biofilm formation and LPS biosynthesis in the treatment group. (**b**) Predicted enzyme differences, with a higher abundance of redox and amino acid-related enzymes in the treatment group.

To investigate the functional consequences of microbial compositional changes, Tax4Fun analysis was used to predict KEGG pathway activity based on 16S rRNA gene sequencing data. As illustrated in Figure 8, significant differences in functional potential were observed between the treatment and placebo groups at both the pathway and enzyme levels. Specifically, predicted relative abundances of pathways associated with microbial virulence and pathogenicity—such as LPS biosynthesis and biofilm formation—were significantly reduced in the PL group (*p* < 0.05), suggesting a decline in microbial pathogenicity following intervention. While pathways related to the biosynthesis of antibiotics and secondary metabolites were elevated in the PL group, core metabolic pathways remained relatively stable. At the enzyme level, five enzymes showed significantly increased predicted abundance in the PL group, including glutathione reductase, nucleoside-triphosphate phosphohydrolase, and various aminotransferases (*p* < 0.05). These enzymes are involved in redox regulation, nucleotide metabolism, and amino acid interconversion, and their upregulation may reflect an enhanced microbial capacity to maintain oxidative balance and metabolic stability. Overall, the Tax4Fun results demonstrate that PL supplementation not only reshaped the taxonomic profile of the oral microbiota but also modulated its functional potential, particularly by downregulating pathogenic pathways and upregulating redox-related enzymatic functions. These changes may contribute to improved host physiological homeostasis.

## 4. Discussion

In this study, we demonstrated that dietary supplementation with a combination of propolis and lysozyme significantly improved oral health in domestic cats with halitosis and exerted modulatory effects on systemic immunity, oxidative stress, and inflammatory responses. These findings highlight a novel strategy for simultaneously enhancing oral and systemic health through targeted modulation of the oral microbiota.

Halitosis is a common clinical problem in domestic cats and is closely associated with oral microbial dysbiosis, plaque accumulation, gingival inflammation, and systemic disorders. Current treatment options include antiseptics such as chlorhexidine [21]; however, chlorhexidine offers only short-term relief of halitosis, the efficacy of chlorhexidine in targeting plaque biofilm is limited, and it may cause adverse effects such as tooth discoloration and taste alteration [22,23]. Natural compounds like catechins have shown promise in reducing halitosis by inhibiting VSC-producing bacteria [10]. In the present study, we observed a rapid and significant reduction in VSC levels in cats receiving PL supplementation, with improvements evident as early as day 14. By day 28, VSC levels in the treatment group showed a clear downward trend toward the baseline levels observed in healthy cats, although a residual statistical difference remained. Mechanistically, polyphenols in propolis—such as gallic acid and quercetin—may chemically neutralize H_2_S via redox mechanisms [24,25], providing a plausible explanation for the observed reduction in oral malodor.

The oral health status of cats can be quantitatively assessed using indices such as the PLI, CI, DI, and GI. Although several natural compounds have been investigated for their potential to improve feline oral health, many exhibit limited efficacy in vivo. For example, while catechins have demonstrated antibacterial effects against biofilm-forming bacteria in vitro, they have failed to significantly improve clinical indicators such as PLI and GI in vivo [10]. Similarly, other natural agents such as *Ascophyllum nodosum* require extended administration periods—often exceeding 90 days—to yield observable clinical benefits [26]. In this study, PL intervention led to significant improvements in GI, PLI, and DI scores within just 14 to 28 days. Notably, by day 28, the treatment group exhibited a 61.04% reduction in GI scores, representing a markedly greater improvement than reported in comparable studies with similar intervention durations. Moreover, the improvement in debris, plaque, and gingival scores in the treatment group reflected a clear trend toward normalization, closely approaching values observed in healthy control cats. This rapid and pronounced effect may be attributed to the complementary actions of propolis and lysozyme. Propolis is rich in flavonoids and phenolic compounds, which possess well-documented antimicrobial, anti-inflammatory, and antioxidant properties [16,17]. Lysozyme enhances mucosal immune stability by hydrolyzing the peptidoglycan layer of bacterial cell walls [12,13]. The synergistic effect of these two compounds may accelerate pathogen clearance, reduce oral inflammation, and promote mucosal recovery, thereby explaining the superior performance of PL relative to other natural substances. These results suggest that PL is a practical and efficient dietary intervention for maintaining feline oral health.

Although the primary focus of this study was the modulation of oral health, the observed systemic improvements imply a broader physiological impact of PL supplementation. Serum concentrations of pro-inflammatory cytokines (TNF-α, IFN-γ) and oxidative stress markers (MDA) were significantly reduced, whereas antioxidant indicators, including SOD and T-AOC, were elevated—collectively reflecting a systemic shift toward immune regulation and redox homeostasis. These effects may be mediated through multiple, potentially overlapping mechanisms. One likely pathway involves the correction of oral dysbiosis, which has been associated with systemic inflammation via the translocation of microbial products or inflammatory mediators from the oral cavity into circulation [27]. Restoration of oral microbial balance has also been linked to systemic anti-inflammatory outcomes in both animal and human studies [28,29]. In parallel, given that PL was administered orally, it is also plausible that its bioactive constituents were absorbed through the gastrointestinal tract and exerted direct systemic effects. Previous studies in larger animals have demonstrated that oral administration of propolis or lysozyme can modulate systemic immune responses and oxidative stress parameters. However, in cats, existing applications of these compounds have primarily focused on localized conditions, such as otitis externa, dermatophytosis, and Cushing’s syndrome, with limited research addressing their systemic effects or underlying mechanisms [30]. This raises the possibility that the systemic benefits observed here may result from both oral cavity and non-oral cavity pathways. Collectively, these results suggest that PL supplementation not only alleviates oral symptoms but also contributes to broader immunological and redox stabilization in feline hosts.

Oral microbiome dysbiosis is a key factor in the development of various oral diseases, including halitosis and gingivitis, and even affects the distal organs through bacterial ectopia [31]. Distinct differences in oral microbial composition exist between healthy cats and those affected by halitosis, underscoring the role of microbiota dysbiosis in disease development. Although research specifically focused on halitosis-related microbiota in cats remains limited, previous studies have characterized significant taxonomic shifts in cats with periodontal disease or gingivostomatitis compared to healthy individuals [32,33,34]. In our study, 16S rRNA gene sequencing of supragingival samples revealed substantial compositional differences between halitotic and non-halitotic cats, as demonstrated by PCoA. These findings confirm that halitosis in cats is associated with disrupted oral microbial communities. Notably, PL supplementation led to a progressive shift in the microbiota of the treatment group toward a taxonomic composition that closely resembled that of healthy controls. This convergence suggests that PL intervention effectively reshaped the dysbiotic oral environment and facilitated the restoration of an oral commensal microbial profile. Such microbial reprogramming may serve as a foundational mechanism for mitigating halitosis severity and sustaining long-term oral health in companion animals.

The reduction in pathogenic bacteria observed in this study may be directly attributable to the antimicrobial and anti-inflammatory properties of propolis and lysozyme. Propolis contains flavonoids, caffeic acid phenethyl ester, and various phenolic compounds, which have been shown to inhibit the growth and virulence of oral pathogens such as *Porphyromonas gingivalis*, *Selenomonas*, and *Desulfovibrio* by disrupting membrane integrity, suppressing protease secretion, and attenuating biofilm formation [35,36]. Lysozyme hydrolyzes the peptidoglycan layer of Gram-positive bacteria and has also been reported to modulate oral biofilm composition [13]. These mechanisms may underlie the observed decrease in *Porphyromonas* abundance (from 32% to 20%), as well as significant reductions in *Selenomonas*, *Desulfovibrio*, and *Peptostreptococcaceae*. *Porphyromonas* is considered a key initiator of halitosis and gingivitis, due to its ability to produce H_2_S, CH_3_SH, and indole, along with multiple virulence factors such as gingival proteases, podocapsules, bacterial fimbriae, LPS, hemolysin, and iron uptake transporters—all of which play crucial roles in bacterial adhesion and host cell invasion [37,38]. Similarly, *Selenomonas* produces VSCs, adheres to periodontal tissues, and releases inflammatory mediators that can promote gingival inflammation [39]. *Desulfovibrio* contributes to H_2_S production via the enzymatic reduction of sulfate through adenosine-5′-phosphosulfate reductase and sulfite reductase pathways [40]. By reducing these pathogens, PL intervention likely disrupted the microbial triggers of halitosis and oral symptoms. In parallel with the reduction in pathogenic taxa, the abundance of commensal genera such as *Moraxella*, *Bergeyella*, and *Flavobacterium* was restored in the treatment group. These genera are typically enriched in the oral microbiota of healthy cats and play crucial roles in maintaining microbial balance [2,41,42].

The correlation analysis between oral bacterial genera and host physiological parameters provided further insights into the mechanisms linking the oral microbiota to both local and systemic health outcomes. Notably, the relative abundances of *Porphyromonas* and *Peptostreptococcaceae* were positively correlated with VSC concentrations and the PLI, which is in accordance with their well-documented roles in VSC production and biofilm formation. Previous studies have established a strong association between *Porphyromonas* and progressive forms of periodontal disease [43], while experimental evidence indicates that targeting *Porphyromonas* can alleviate halitosis and oral inflammation [10]. In our study, PL intervention resulted in a significant reduction in *Porphyromonas* abundance within the gingival microbiota of halitotic cats, compared to the placebo group. This microbial shift may represent a key mechanism underlying the observed improvement in halitosis and gingival inflammation. On the other hand, commensal bacteria such as *Moraxella* and *Flavobacterium* were negatively correlated with VSC concentrations and oral symptom indices. Previous studies have shown that in healthy cats’ full niche sampling, *Moraxella* is the second most abundant genus after *Unclassified Pasteurellaceae* [41]. Post-treatment improvements in oral health among cats and dogs have frequently been accompanied by increased relative abundances of *Moraxella* and *Luteimonas* [2,44,45]. These commensal genera may contribute to the maintenance of oral microbial balance and indirectly relieve clinical symptoms of halitosis and gingivitis.

Additionally, *Porphyromonas* and *Fusobacterium* were positively correlated with circulatory oxidative stress markers such as MDA and pro-inflammatory cytokines, including TNF-α and IFN-γ. Previous studies have demonstrated that both genera secrete virulence factors that stimulate host immune responses, leading to elevated levels of reactive oxygen species (ROS) and inflammatory mediators [46,47]. Since the oral cavity is the starting point of the digestive system, pathogenic bacteria may disseminate from the oral cavity and have an impact on distant organs. It has been estimated that 10^11^ oral bacteria make their way through the digestive system each day [48]. This contributes to chronic low-grade inflammation and the development of systemic diseases such as cardiovascular disease and diabetes [47,49]. However, commensal bacteria including *Moraxella*, *Luteimonas*, and *Flavobacterium* were negatively correlated with inflammatory markers and positively correlated with antioxidant enzymes such as SOD and T-AOC. These associations suggest that certain beneficial bacteria may contribute to systemic redox balance and immunomodulation, potentially through host–microbe interactions or the production of anti-inflammatory microbial metabolites. Furthermore, the observed associations between *Bergeyella* and *Streptococcus* with IgG levels may reflect a role in mucosal immune activation [50]. Although causality cannot be confirmed, these results collectively support the notion that restoration of oral microbiota composition may exert systemic effects, particularly in modulating oxidative stress and immune homeostasis.

Functional predictions derived from Tax4Fun analysis further indicate that PL supplementation reshaped key microbial functional capacities relevant to both oral and systemic health. At the pathway level, several virulence-associated processes were downregulated in the treatment group, including LPS biosynthesis and biofilm formation. These pathways are known to enhance microbial pathogenicity and stimulate host inflammatory responses by activating toll-like receptor signaling and promoting microbial persistence through biofilm development [51]. Meanwhile, the predicted biosynthesis of antibiotics and secondary metabolites was significantly upregulated following PL intervention. Recent studies suggest that commensal bacteria may enhance secondary metabolite production under ecologically stable conditions to support microbial community resilience and suppress opportunistic pathogens [52]. The increased abundance of these pathways may thus reflect the emergence of a functionally enriched, commensal microbiome capable of contributing to microbial homeostasis and immunological balance. At the enzyme level, PL supplementation significantly enhanced predicted microbial expression of enzymes such as glutathione reductase, aryl-alcohol dehydrogenase, and multiple aminotransferases. Glutathione reductase plays a key role in regulating intracellular GSH–GSSG ratios, a critical determinant of microbial and host redox balance [53]. Aminotransferases are involved in nitrogen and carbon metabolism, essential for amino acid homeostasis and microbial adaptation to oxidative or inflammatory stress [54]. These results suggest that, beyond taxonomic restructuring, PL intervention reprograms microbiome function in ways that may confer both intraoral and systemic benefits, supporting the hypothesis that natural compounds such as propolis and lysozyme exert their effects through functional microbial modulation.

This study has several limitations, including a relatively modest sample size and a short intervention duration. Future investigations should incorporate extended follow-up periods to evaluate potential long-term effects on alveolar bone integrity and plaque mineralization. Additionally, several oral bacterial taxa identified in this study were significantly correlated with systemic antioxidant and inflammatory markers, suggesting a potential mechanistic link between oral microbiota modulation and host systemic physiology. However, because PL was administered via oral ingestion, it remains unclear whether the observed systemic improvements were primarily mediated through local microbial restructuring or via direct gastrointestinal absorption of bioactive compounds. These findings raise the possibility that PL supplementation may confer integrated systemic benefits beyond oral health alone. As blood samples from healthy cats were not collected due to ethical considerations, systemic improvements were evaluated based on between-group comparisons. To further elucidate the underlying mechanisms, future studies employing localized delivery strategies (e.g., oral sprays or gels) will be essential to distinguish the respective contributions of local and systemic pathways.

## 5. Conclusions

In summary, this study demonstrates that dietary supplementation with a combination of propolis and lysozyme significantly improves oral health in domestic cats with halitosis by reducing VSC levels, gingival inflammation, and plaque accumulation. PL intervention not only altered the taxonomic composition of the oral microbiota but also restored a commensal microbial profile and enhanced functional pathways related to antioxidant activity and reduced microbial virulence. Moreover, systemic improvements—including reduced oxidative stress and inflammatory markers—were observed, suggesting that PL may exert both local and systemic benefits. These findings highlight the potential of PL as a practical, natural, and effective dietary strategy for managing feline oral health and possibly influencing systemic physiological balance. Further research with larger sample sizes and extended intervention durations is warranted to clarify the long-term effects of PL supplementation. In particular, studies employing germ-free animal models or metabolomic profiling are recommended to dissect the relative contributions of local (oral microbiota modulation) versus systemic (gastrointestinal absorption) pathways underlying the observed physiological improvements.

## Figures and Tables

**Table 1:** Demographic data of experimental cats.

	Health Control Group	Placebo Group	Treatment Group	*p*-Value
Number	8	8	8	
Gender (Male/Female)	4/4	4/4	4/4	
Age (month; mean ± s.d.)	12.65 ± 3.02	13.25 ± 1.91	13.38 ± 2.5	0.8174
Weight (kg; mean ± s.d.)	3.00 ± 0.78	3.08 ± 0.83	2.84 ± 0.9	0.8481
VSC concentration (ppm; mean ± s.d.)	332.38 ± 56.22	861.67 ± 14.97	858.33 ± 165.23	0.9669 (P vs. T)
				0.0000 (HC vs. P and T)

HC, health control group; P, placebo group; T, treatment group.

## Data Availability

The author confirms that all data generated or analyzed during this study are included in this published article.

## Abbreviations

The following abbreviations are used in this manuscript:

PL	Combining propolis and lysozyme
VSCs	Volatile sulfur compounds
HC	Health control group
PLI	Turesky–Gilmore–Glickman modification of the Quigley–Hein plaque index
CI	Ramfjord calculus index
GI	Modified Loe and Silness gingival index
DI	Greene–Vermillion debris index
IgA	Immunoglobulin A
IgG	Immunoglobulin G
IgM	Immunoglobulin M
MDA	Malondialdehyde
SOD	Superoxide dismutase
T-AOC	Total antioxidant capacity
CAT	Catalase
GSH-PX	Glutathione peroxidase
TNF-α	Tumor necrosis factor-α
IL-2	Interleukin-2
IL-4	Interleukin-4
IFN-γ	Interferon-γ
PCoA	Principal coordinate analysis
LEfSe	Linear discriminant analysis effect size
LDA	Linear discriminant analysis
